# Urea intercalated encapsulated microalgae composite hydrogels for slow-release fertilizers

**DOI:** 10.1038/s41598-024-58875-1

**Published:** 2024-07-01

**Authors:** Nada Sarhan, Esraa G. Arafa, Nada Elgiddawy, Khaled N. M. Elsayed, Fatma Mohamed

**Affiliations:** 1 Department of Biotechnology and Life Sciences, Faculty of Postgraduate Studies for Advanced Sciences (PSAS), Beni-Suef University, Beni-Suef, 62 511 Egypt; 2 Chemistry Department, Faculty of Science, Beni-Suef University, Salah Salim St., Beni-Suef, 62514 Egypt; 3 Botany and Microbiology Department, Faculty of Science, Beni-Suef University, Beni-Suef, 62511 Egypt; 4 Materials Science Research Laboratory, Chemistry Department, Faculty of Science, Beni-Suef University, Beni-Suef, 62514 Egypt; 5 Nanophotonics and Applications Lab, Faculty of Science, Beni-Suef University, Beni-Suef, 62514 Egypt

**Keywords:** Microalgae, Hydrogels, Slow-release fertilizer, Water retention, Environmental sciences, Chemistry, Materials science

## Abstract

In agriculture, hydrogels can be addressed for effective operation of water and controlled-release fertilizers. Hydrogels have a significant ability for retaining water and improving nutrient availability in soil, enhancing plant growth while reducing water and fertilizer usage. This work aimed to prepare a hydrogel composite based on microalgae and biopolymers including chitosan and starch for use as a soil conditioner. The hydrogel composite was characterized by FTIR, XRD, and SEM. All hydrogel properties were studied including swelling degree, biodegradability, water-holding capacity, water retention, and re-swelling capacity in soil and water. The urea fertilizer loading and releasing behavior of the prepared hydrogels were investigated. The results revealed that the range of the maximal urea loading was between 99 and 440%, and the kinetics of loading was fitted with Freundlich model. The urea release % exhibited 78–95%, after 30 days, and the kinetics of release was fitted with zero-order, Higuchi, and Korsmeyer–Peppas models. Furthermore, the prepared hydrogels obtained a significant water-holding capacity, after blending soil (50 g) with small amount of hydrogels (1 g), the capacity increased in the range of 99.4–101.5%. In sum, the prepared hydrogels have the potential to be applied as a soil conditioner.

## Introduction

The provision of adequate amounts of water and fertilizer in agricultural production is the challenge for achieving optimal plant growth^1^. Unfortunately, the introduction of chemical agriculture including chemical fertilizers into the soil enhances the crop production^2^. However, owing to volatilization, leaching and denitrification, a lot of N fertilizer is lost to the environment and lead to increase the nitrate and N_2_O (greenhouse gases) levels in groundwater^3^. Following the loss of fertilizer, plants are deprived of nutrients and the environment is harmed^4,5^. In addition, most water that applied in irrigation was consumed one as a result of high evaporation, low rainfall and high variability^6^. Consequently, fertilizer and water supplies consider the two main factors influencing crop growth and also essential for sustainable agricultural development^7^. Thus, it is crucial to design a suitable material with an effective water-holding capacity and controls fertilizer-release^8,9^.

Bio hydrogels are extremely absorbent natural polymers that can keep water and nutrients in the soil around a root plant when it starts to dry out^10^. Hydrogel has been applied for enhancing the water retention of soil and water-managing materials for degraded land^1^. It is possible to define hydrogels as hydrophilic polymeric materials with network structures and the proper amount of cross-linking. Hydrogel can hold large amounts of water even under pressure. Slow release fertilizer (SRF) can improve the consumption of nutrients and reduce crop and environmental damage, according to many literatures^4,11–14^. Besides, the evaporation loss and the frequency of water irrigations were declined^15^. When fertilizer is incorporated into the hydrogel, slow release fertilizer hydrogel (SRFH) is produced which can enhance water retention in soil^16^and control the fertilizer release which in turn to enhance the plant growth^17,18^.

Hydrogels can be prepared via cross-linked of biodegradable polysaccharides^19–21^. Among them, starch and chitosan had unique properties such as; biodegradability, biocompatibility, lack toxicity, low cost, and easy gelation^22–25^. The hydrophilic group of starch (e, g., –OH), diffused in the molecular chains which facilitate the biodegradability of hydrogels^26,27^. Chitosan is obtained by partial deacetylation^28^ of insoluble naturally chitin, from exoskeletons of crustaceans, fungi, and insects^29,30^. Chitin has a rigid crystalline structure due to hydrogen interactions between acetamide groups and hydroxyl groups^31^. Because of its high concentration of acetylated groups, stiff structure, and low solubility in aqueous solutions, chitin is not easily applied. A partial deacetylation of chitin results in chitosan, which has more amino groups and is more soluble in water. Increasing the degree of chitosan deacetylation improves the material's biocompatibility and biodegradability^14,32^.

Microalgae have significant advantages for plant growth and are a rich supply of lipids, proteins, carbohydrates, pigments, and vitamins^33^. In earlier research, hydrogels were employed to immobilize microalgae^34^. The most recent successful 3D bio printing of cell-friendly hydrogel structures, such as the creation of hydrogel scaffolds loaded with photosynthetic algae^35,36^. It has been shown in a lab setting with specialized apparatus^37^. Though it has been the subject of much less research, the microalgal biomass can be employed as an organic slow-release fertilizer^38^. Microalgae can serve as an organic fertilizer to stop nutrient losses by releasing N, P, and potassium (K) gradually based on plant requirements^39^.

As a soil conditioner, hydrogel enhances the retention of the soil for nutrients and water and serves as an agent for slow-release fertilizers^40^. Introducing the hydrogels to the soil improves soil density, structure, and permeability, which in turn boosts the water infiltration and evaporation rates. However, the water run-off and erosion decrease increases crop productivity and practicable crop yield^41^.

This study introduces the first modification of Microalgae hydrogel using different biopolymer including chitosan and starch for using as a soil conditioner. The structure and composition of the produced hydrogels nanostructures were evaluated using FT-IR, X-ray diffraction, and scanning electron microscopy. In addition, the loading and release of urea mechanism using bio hydrogel were confirmed through SEM technique. The potential use of these hydrogels as controlled-release fertilizers (CRFs), water retention (WR), and water-holding capacity (WHC) in soil were evaluated.

## Materials and experimental part

### Materials

Chitosan (CS; Bio Basic, ON, Canada) with a deacetylation degree of 96%, Urea (Piochem, Egypt), and starch extra pure potatoes (St; Piochem, Egypt). *Synechocystis* sp. PAK 13 (Sy) was isolated from the soil and water in Egyptian environments, Beni-suef habitats and marine habitat (Red Sea) and made the molecular characterization by Elsayed et al.^42^. The selected strain were obtained from Microbiology Department, Faculty of Science Beni-Suef University, Egypt and then cultivated as it is without modified on 1 ml Wuxal media (WM) with 1 l distilled water and 5 g sodium chloride in microbiology lab, bani-suef university, Egypt.

### Experimental

#### Preparation of chitosan/strach/microalgae-based hydrogel

**Figure 1:**
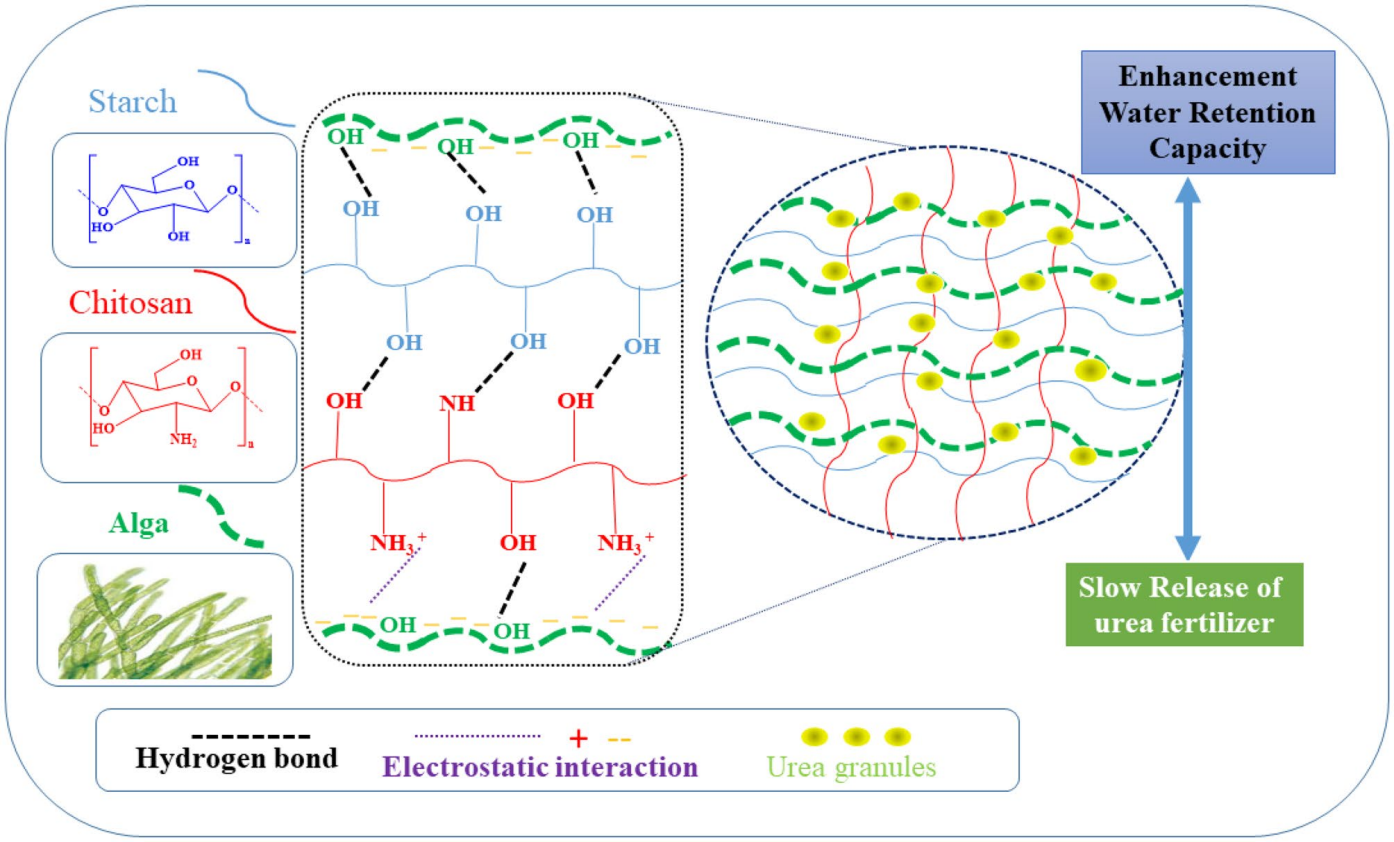
Schematic diagram illustrates the basic idea.

At room temperature, chitosan (CS) was dissolved in 25 ml of 1% aqueous acetic acid solution. Starch (St) solution was dissolved in 25 ml of distilled water and mechanically stirring it for 20 min at 70 °C. Finally, the algae solution (Sy) was suspended in 25 ml (50:50 V) distilled water and ethanol and mechanically stirred it for 1 h at 70 °C. All CS, St, and algae solutions were mechanically stirred together for 1.5 h at room temperature. The hydrogels were dried at 40 °C for 24 h (Fig. 1). Table 1 represents the codes of the prepared CS, St, and Sy hydrogel samples.

**Table 1:** Samples codes.

Sample code	CS	St	Sy
CS:St:Sy (Sy-St-CS)	10	10	1
CS:Sy (Sy-CS)	10	–	1
St:Sy (Sy-St)	–	10	1

#### Hydrogel characterizations

Several methods were used to characterize the hydrogels that were unloaded and loaded with urea: the crystallinity is determined by X-ray diffraction (XRD, PANalytical Empyrean, Sweden), which uses an X-ray diffractometer with Cu-Kα radiation (wavelength 0.154 nm) operating at a current of 35 mA and voltage of 40 kV, scanning at a pace of 8° min^−1^ from 5° to 80° (2θ). Functional group vibration is examined using Fourier transform infrared spectroscopy (FTIR; Bruker-Vertex 70, high sensitivity DLaTGS with KBr pellet technique, Germany) at 400–4000 cm^−1^. To describe the surface morphology of hydrogels, a field emission scanning electron microscope (FESEM, Zeiss Sigma 500 VP, Germany) is utilized, and samples were obtained by adding a little part of the film on a carbon tube on a stub, which was coated with a thin layer of gold.

#### Swelling measurements and kinetics

In distilled water, the hydrogel swelling rate and steady-state swelling ratio were determined. This method has been used by other researchers^43,44^. In order to conduct this study, samples of 0.1 g dried hydrogel (W_0_) were submerged in 25 ml of distilled water at various intervals (15–300 min) at room temperature. To remove extra surface water, the outer surface of each hydrogel was lightly dried with filter paper after being sieved out of the water. The hydrogel was then weighed (W_s_), and the weight change was noted. Using Eq. (1), the percentage of the swell ability (swelling degree%) was calculated as follows^45^:

1 $$\mathrm{Swelling\, degree\% }= (\frac{{w}_{s}- {w}_{0}}{{w}_{0}})\times 100$$

where W_s_: is the weight of the hydrogel when it is swollen; W_0_: is the dry hydrogel's weight.

#### Water-holding capacity (WHC) in soil

WHC was used to measure how well the prepared hydrogel retained water in the soil. The soil was dried at 45 °C in this experiment until it reached a constant weight. 50 g of the dried soil and 1 g of dried hydrogels were thoroughly combined before being placed in a glass pot (W_1_). The control (W_0_) was made up of only pure soil. After weighing each pot, 5 ml of distilled water was added, and the weight was then measured once more (W_2_). Equation (2) was used to calculate the WHC as follows^46^:

2 $$\mathrm{WHC\% }= \frac{({W}_{2}-{W}_{1})}{{W}_{0}}\times 100$$

#### Water retention (WR)

After that, the samples from the WHC study were kept at RT, and the weight was checked every day until no discernible weight loss was seen. Equation (3) below gave values^47^:

3 $$\mathrm{WR\% }= \frac{{W}_{t} -\mathrm{ W}}{{W}_{i} - W}\times 100$$

where W is the sample's weight before adding water, W_i_ is the sample's weight after doing so, and W_t_ is the sample's weight at various time intervals.

#### Reswelling capacity (RSC) in soil and water

In media and soil, the hydrogels' RSC capacity was assessed. As mentioned above for swelling (“Hydrogel characterizations”), in the first of the two methods, a predetermined amount of the hydrogel was swollen in distilled water until equilibrium had been reached. The swollen hydrogels were then dried at 40 °C until there was no weight variation, after which they were placed back into distilled water until equilibrium was restored. The RSC in soil was examined in the ways listed below. The WHC study's (“Swelling measurements and kinetics”) moist soil samples were dried at 45 °C to a constant weight. The samples were then reweighed after 50 ml of distilled water had been slowly added to each pot. For each hydrogel sample, this process of swelling, drying, and swelling was repeated five times in order to assess the hydrogel's reversibility and water absorption capacity. Equation (1) for the swelling given above was used to calculate the RSC (in percent) of the hydrogel for each reswelling cycle.

#### Biodegradation test

The prepared hydrogels were subjected to soil burial biodegradation testing using agricultural soil. To prevent water loss through evaporation, a portion of the prepared hydrogel (0.1 g) was buried in soil (200 g) at a depth of about 2–3 cm in pots kept at room temperature under moisture-controlled conditions. The sample was placed in the soil and irrigated every three days. It was then taken out of the soil every six days, dried in a vacuum oven and weighed. Calculating the weight loss of the hydrogels allowed researchers to examine the severity of degradation at each stage. Equation (4) was used to calculate the percentage of weight loss (degradation rate) as follows^48^:

4 $$\mathrm{Weight\, loss\% }= \frac{{W}_{i}-{W}_{f}}{{W}_{i}}\times 100$$

where W_i_ is the hydrogels' initial weight and W_f_ is their final weight after six days, respectively.

#### The loading capacity of hydrogels

Different urea concentrations (2–9 M) were used to calculate the hydrogel's loading capacity. For 1.5 h at room temperature, 0.1 g of the dry hydrogel was dissolved in 20 ml of urea fertilizer solution. After the solution had swelled, it had been filtered, and the hydrogel had been dried at 40 °C for 24 h (Wt_f_).

The loading % was calculated using the following equation^49^:

5 $$\mathrm{Urea\, loading \% }= \frac{ {Wt}_{f}-{Wt}_{0}}{{Wt}_{0}}\times 100$$

where Wt_f_ is the loaded hydrogel's weight and Wt_0_ is the unloaded hydrogel's weight.

The absorbance of the filtrate was measured by UV-Spectrophotometer a double beam spectrophotometer (Perkin Elmer, Lamba 950, Waltham, USA) was used.

At ℷ_max_ = (296 nm).

#### Urea release test

Distilled water was used as the release medium to measure the release of urea from the hydrogel. Loaded hydrogel (0.2 g) was only in a 100 ml Erlenmeyer flask of distilled water at room temperature. 1 ml was aspirated at RT intervals, and the absorbance at 296 nm was calculated. To maintain the same volume, 1 ml of distilled water was added to replace the aspirated solution. The following Eq. (6) were used to calculate the urea release percentage^50^:

6 $$\mathrm{Release\% }= ({\text{conc}}.\mathrm{ of \,released \,urea}/\mathrm{urea \,conc}.\mathrm{in \,the \,hydrogel}) \times 100$$

## Results and discussion

### Characterization

#### FT-IR

**Figure 2:**
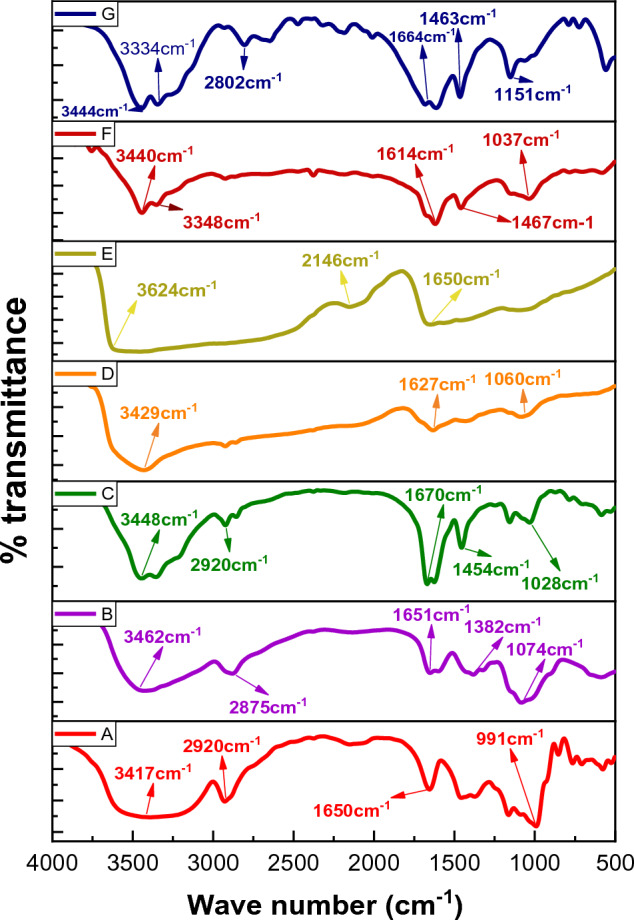
FTIR spectrum of (**A**) Starch St, (**B**) Chitosan CS, (**C**) Microalgae Sy, (**D**) Sy-St hydrogel, (**E**) unloaded Sy-St-CS hydrogel, (**F**) urea-loaded Sy-St-CS hydrogel, and (**G**) urea.

FT-IR spectra of St, CS, Sy, Sy-St, bare Sy-St-CS hydrogel, urea-loaded Sy-St-CS hydrogel, and urea were shown in Fig. 2A–G. The absorption peaks of St were characterized by the following bands: the band at 3417 cm^−1^ was due to O–H stretching vibration. The peak appeared at 2920 cm^−1^ was ascribed to C–H asymmetric stretching. While the peaks appear at 1650 and 991 cm^−1^ due to C–H bending and C=C bending, respectively^51^. CS spectrum showed a broad band at 3462 cm^−1^, and 2875 cm^−1^ corresponding to the stretching vibrations of O–H and N–H groups, C–H stretching, respectively. Also exhibited peaks at 1074 cm^−1^, 1651 cm^−1^, and 1382 cm^−1^ corresponding to C–N stretching, (C=O amide) stretching, and stretching vibration of C–N bond, respectively^52,53^. However, Sy spectrum showed five peaks at 3448 cm^−1^, 2920 cm^−1^, 1670 cm^−1^, 1454 cm^−1^, and 1028 cm^−1^ corresponding to O–H stretching, N–H stretching, C–H stretching, C=O stretch vibrations, and C–O stretching, respectively.

Sy-St hydrogel spectrum displayed three peaks at 3429 cm^−1^, 1627 cm^−1^, and 1060 cm^−1^ corresponding to is assigned stretching vibration of the –OH. C=O stretching, C–O–C, respectively. Sy-St-CS hydrogel spectrum showed three significant peaks at 3624 cm^−1^, 2146 cm^−1^, and 1650 cm^−1^ for O–H stretching, and C=N stretching, respectively.

Urea spectrum appeared two bands at 3444 cm^−1^, and 3334 cm^1−^ for N–H stretching (primary NH_2_), 1664 cm^−1^ for C=O stretching, and 1463 cm^−1^ for –C=N. Urea-loaded Sy-St-CS hydrogel spectrum has two bands at 3440 cm^−1^ and 3348 cm^−1^ for –NH stretching of urea, and another band appear at 1467 cm^−1^ for C=N stretching of urea which proved the good loading of urea.

#### Structural properties of hydrogel

**Figure 3:**
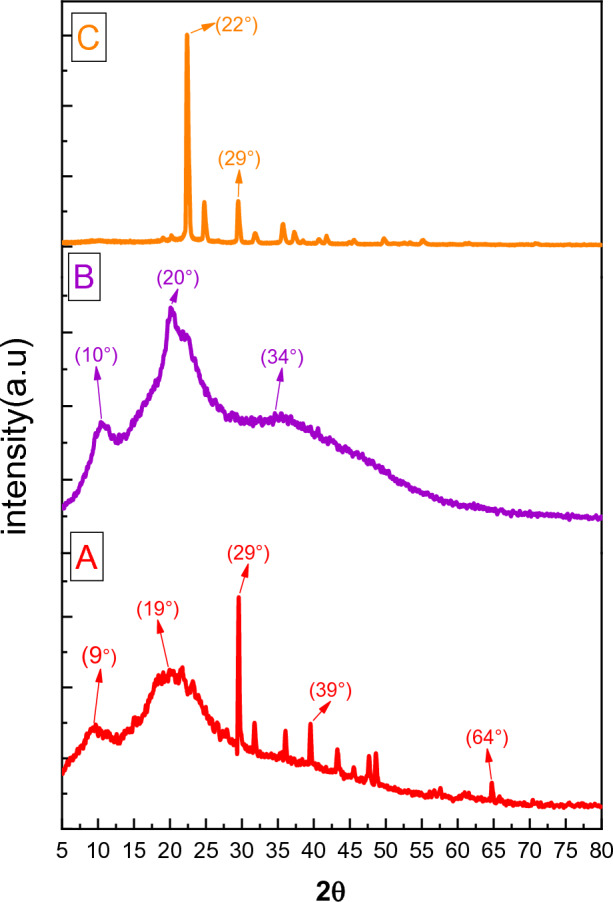
XRD of (**A**) Microalga Sy, (**B**) unloaded Sy-St-CS hydrogel, and (**C**) urea-loaded Sy-St-CS hydrogel.

XRD spectra of Sy, bare Sy-St-CS hydrogel, and urea-loaded Sy-St-CS hydrogel are shown in Fig. 3. The XRD pattern of Sy appeared with sharp crystalline peaks at around 2θ = 29°, 31°, 36°, 39°, 43°, 47°, and 64°, and two broad peaks at around 2θ = 9° and 19°, which confirmed its crystalline nature, and the crystallite size was calculated through Braggs’ equation, was 365.56 Å. While the XRD pattern of the unloaded hydrogel showed a peak at 20°, which represented chitosan, the crystalline peaks decreased compared to the Sy chart, which was illustrated due to the considerable decrease in its crystallinity following incorporation within the hydrogel^54^.

In comparison, the XRD pattern of the urea-loaded Sy-St-CS hydrogel showed sharp peaks showing high crystallinity and indicating the appearance of formed urea crystals on the surface at around 2θ = 22°^55^. In addition to the presence of two sharp crystalline peaks at around 2θ = 24° and 35° due to starch hydrogel^56^. Also, the crystallite size of the urea-loaded Sy-St-CS hydrogel increases compared with the unloaded hydrogel, where in the case of the unloaded hydrogel is 110.12 Å, whereas that of the loaded hydrogel is 147.67 Å.

#### Morphological properties for hydrogels

**Figure 4:**
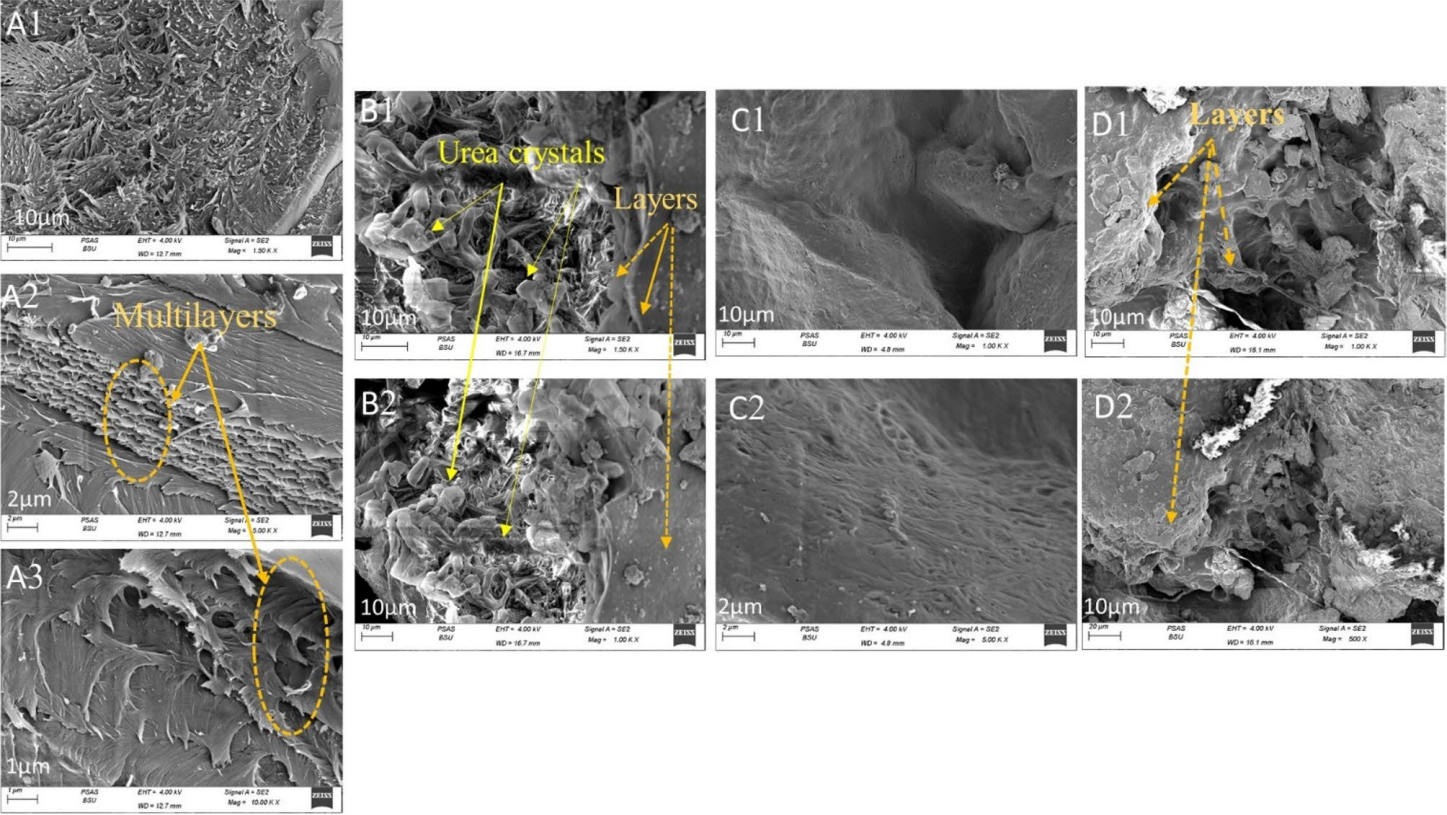
SEM of (A1-3) unloaded Sy-St-CS hydrogel, (B1-2) urea-loaded Sy -St-CS hydrogel, (C1-2) urea-release, and (D1-2) biodegradation of Sy-St-CS hydrogel.

The morphological surface of the bare hydrogel showed an unsmooth surface with cavities and multilayers that were stacked as sheets, as represented in Fig. 4A (1–3). There are cavities between layers that are able to accommodate and absorb the urea crystal between them. Thus, the surface morphology of urea-loaded Sy-St-CS hydrogel was filled with urea crystals in those cavities between hydrogel layers, as shown in Fig. 4B(1–2), which proved the good loading of urea on hydrogel. While the release of urea in water by Sy-St-CS hydrogel after 30 days is represented in Fig. 4C(1–2), Its surface was bare and etched without urea crystals compared to the loaded surface of Sy-St-CS hydrogel, and the layered nature of hydrogel was lost as a result of the release of urea crystals from them. Interestingly, the stability of the Sy-St-CS hydrogel was confirmed through SEM in Fig. 4C(1–2). The layered nature of hydrogel remained after 30 days in the soil. There are slight differences as compared to the unloaded Sy-St-CS hydrogel surface, as shown in Fig. 4D (1–2).

### Swelling degree of hydrogel samples

**Figure 5:**
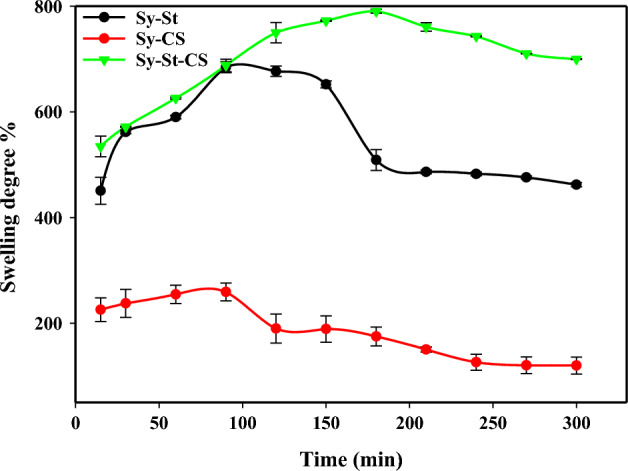
The swelling behavior of hydrogel samples (Sy-St, Sy-CS, Sy-St-CS) with different time intervals.

Figure 5 shows the water absorption by three hydrogels (Sy-St, Sy-CS, and Sy-St-CS hydrogels) at various time intervals (15–300 min). It was observed that all hydrogels initially absorbed a significant amount of water and then slightly increased over time. For up to 90 min, the maximum swelling ratio was noted. The composition of the hydrogels is mainly based on microalgae for its ability to absorb water and its many benefits to the plant according to its hydrophilicity, biocompatibility, and ease of utilization. The encapsulation of microalgae with polymers increases the ability of the hydrogel to absorb distilled water due to its hydrophilic nature. The highest initial swelling rate was found at Sy St-CS hydrogel percent of 688%, which was attributed to the presence of three different polar functional groups (OH, NH_2_, and C–O–C) related to chitosan and starch, beside algae, which have high water capacity^57–59^. The solvent molecules are absorbed into the three-dimensional network in the hydrogel due to the appearance of polysaccharides, hydrophilic groups, electrostatic repulsions of the amphiprotic groups, and hydrogen bonds in the polymeric chains^57,60^. The hydrogen bonds formed by the OH groups in starch, chitosan, and algae are the main cause of their high hydrophilic properties and strong intermolecular attraction^14,58^. At Sy-St hydrogel, the swelling percent degree was 684%. This significant ratio was due to the large numbers of OH groups that can be found in the structures of starch and algae. Each glucose unit in starch contains one of two different types of OH groups. These OH groups have a significant impact on the reactivity of starch with algae^59,61^. While in Sy-CS hydrogel, the swelling percent was 259%, which has the lowest swelling rate due to less hydrogen bonding than others. After 150 min, we noticed that the swelling rate decreased in the Sy-St hydrogel. This decline may be attributed to the hydrogels bond breaking and becoming brittle due to water excess^62^. The fast swelling and high sorption characteristics may be considered as strong evidences for such materials to behave as superabsorbent materials. Once the equilibrium is reached, de-swelling phenomena of hydrogels was occurred. De-swelling is the release of absorbed water by the hydrogels. As expected, for the hydrogels with higher absorbency, the de-swelling ratio was also higher^63^. All the samples showed de-swelling phenomena, moreover Sy-St hydrogel showed the higher deswelling degree that showed higher swelling degree.

### Loading properties and mechanism

**Figure 6:**
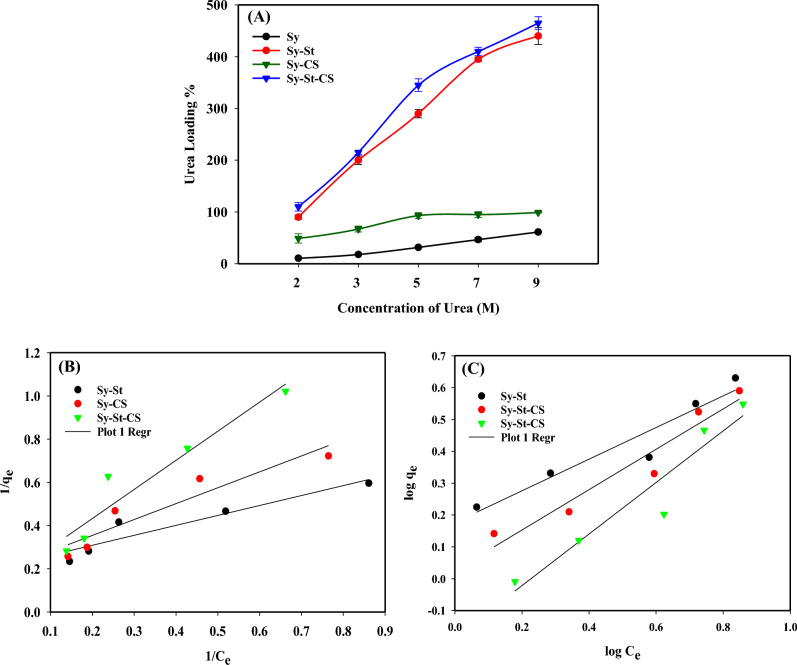
Loading properties and mechanism of the hydrogels sample (Sy-St; Sy-CS; Sy-St-CS). (**A**) Loading percentage of urea on hydrogels samples, (**B**) fitting of the loading results with Langmuir model, and (**C**) fitting of the loading results with Freundlich model. Suppl. Figure 1: ANOVA residual plots for urea loading through Sy-St-CS hydrogel.

Generally, the degree of hydrogel swelling determines loading capacity. The aqueous solutions with various urea concentrations (2–9 M) are shown in Fig. 6A. The equilibrium time and various urea concentrations provide important details regarding the hydrogels' maximal loading capacity, and their equilibrium models are essential for comprehending the primary loading mechanism^64^. As the concentration of urea was increased, the loading increased until it reached equilibrium or saturation at 9 M, with clear preferences for the Sy, Sy-St, Sy-CS, and Sy-St-CS hydrogel samples. It is anticipated that there will be a direct correlation between swelling degree and loading capacity. According to the high observed equilibrium swelling degrees for Sy-St-CS hydrogel percent, this hydrogel has a greater loading capacity than others. The sub-maximum fertilizer loading at the Sy-St hydrogel percentage was 44%, and the least loading rate was found at the Sy-Cs hydrogel percentage of 99%. Additionally, the loading percentage of the algae alone was lower than the hydrogels, which was 61.2%. By using the theoretical equilibrium models of Langmuir and Freundlich, it was possible to ascertain whether the loading of urea onto the hydrogels produced monolayer or multilayer absorption. The Freundlich model depicts the heterogeneous adsorption system of multilayer absorption types^65^, while the Langmuir model describes the homogeneous monolayer adsorption system types that occur within the receptor sites at the surface of the adsorbent^66^. When discussing the absorption between the liquid and soil phases, both models are typically employed. By mathematically fitting the Langmuir and Freundlich equations, the outcomes of the Langmuir and Freundlich isotherm models were achieved^67^. An expression for the Langmuir equation is as follows:

7 $$\frac{{c}_{e}}{{q}_{e}}=\frac{1}{{q}_{0}b}+\frac{{c}_{e}}{{q}_{0}}$$

where the equilibrium concentration of urea is c_e_ (mg/l). The amount of urea adsorbed per g of the adsorbent at equilibrium is known as q_e_ (mg/g). The highest monolayer adsorption rate is Q_0_ (mg/g). The Langmuir constant, which represents the energy of adsorption, is b (l/mg).

The empirical Freundlich equation is expressed as follows:

8 $$log {q}_{e} =log {K}_{f} +\frac{1}{n}{c }_{e}$$

where q_e_ (mg/g) is the quantity of urea adsorbate that has been absorbed for every gramme of adsorbent. The equilibrium urea concentration is represented by c_e_ (mg/l). K_f_, and n are the Freundlich isotherm constants that signify, respectively, adsorption capacity and intensity^68^.

The Langmuir and Freundlich parameters are shown in Table 2. The loading behavior of urea by Sy-CS and Sy-St hydrogels appeared to be of excellent fitting with Freundlich model, as shown in Fig. 6B,C and Table 2. It was clear from the R^2^ values and linearity of graph in the two hydrogels that urea adsorption took place as a heterogeneous multilayer adsorption. However, Sy-St-CS hydrogel had approximate R^2^ values of 0.916 and 0.915 for the Freundlich and Langmuir models, respectively. This indicates that the urea was adsorbed by surface adsorption with multilayer adsorption between their layers as a result of strong absorbate–absorbate interaction^69^.

**Table 2:** Represent the equations of isotherm models and parameters from the hydrogels samples.

Modal	Parameters	Sy -St	Sy -CS	Sy-St-CS
Langmuir	Q_0_ (mg/g)	4.619	4.826	6.130
	K_L_ (L/mg)	2.129	3.550	8.257
	R^2^	0.888	0.886	0.916
Freundlich	K_f_ (mg/g)	1.499	1.061	0.656
	N	2.013	1.579	1.236
	R^2^	0.916	0.933	0.915

### Urea release behavior

**Figure 7:**
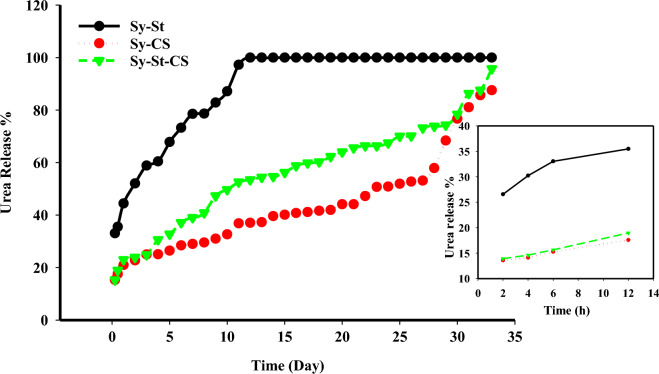
Urea release percentage from Sy-St, Sy-CS, and Sy-St-CS hydrogels, inset showing the release percentage at initial hours for three samples.

All hydrogels were tested to examine their urea release characteristics in water at room temperature. The urea release behavior through three loaded hydrogels (Sy-CS, Sy-St, and Sy-St-CS) was investigated. According to Fig. 7, it is noticed that high releasing rate during the first five hours, followed by a low releasing rate from day 2 to day 29 and then reached nearly equilibrium curve from day 30 to day 33 in case of Sy-CS, and Sy-St-CS hydrogels. However, Sy-St hydrogel, the urea release behavior exhibit a high releasing rate during the first five hours, followed by a low releasing rate from day 2 to day 11 and then total urea release occurred from day 12 to day 33. This could be related to weakly bonded urea onto the external surface of Sy-St hydrogel by weak hydrogen bonding, which facilitated their sudden diffusion and dissolution into the solution^70^. Therefore, the nature of the loading process may be to blame for the urea release behavior. In the case of Sy-St hydrogel, urea absorption took place, and the release percentage reached 44% on the first day, 58% after three days, and 100% after 11 days for the urea loaded. For samples of the hydrogels Sy-CS and Sy-St-CS, the release percentages on the first day were 15% and 15%, respectively, and on the third day, they were 24% and 24%, respectively. Additionally, the cumulative release percentages for Sy-CS and Sy-St-CS hydrogel were 87% and 95%, respectively, after 30 days.

#### Kinetic study of urea release

The urea fertilizer kinetic release behavior of Sy-CS, Sy-St, and Sy-St-CS was investigated using zero-order, first-order, Higuchi, Hixson–Crowell, and Korsmeyer–Peppas models. The first-order and zero-order models are examples of common kinetic models. The zero-order model^71^ states that the releasing rate happened at a fixed pace over a predetermined amount of time and was independent of fertilizer concentration. The releasing rate, which was controlled by fertilizer content and happened at a constant rate, was examined using the first-order model. Table 3^72^ could be used to display the representative equations of the zero-order and first-order kinetic models. But the releasing behaviour of fertilizer being dissolved and spread from hydrogel systems was explained by the Higuchi model^73^.

**Table 3:** Represent the Equations of kinetic models and parameters from the hydrogels samples.

Kinetics modal	Equations	Sample code	R^2^
Zero order	W_0_ − W_t_ = K_0_t	Sy-St	0.717
		Sy-CS	0.893
		Sy-St-CS	0.957
First order	logW_t_ = logW_0_ + $\frac{{K}_{1t}}{2.303}$	Sy-St	0.819
		Sy-CS	0.708
		Sy-St-CS	0.799
Higuchi model	W_t_ = K_h_t^1/2^	Sy-St	0.829
		Sy-CS	0.801
		Sy-St-CS	0.969
HixsonñCrowell model	${{W}_{0}}^{1/3}-{{W}_{t}}^{1/3}= {K}_{HCt}$	Sy-St	0.927
		Sy-CS	0.779
		Sy-St-CS	0.903
Korsmeyer–Peppas model	$\frac{{w}_{t}}{{w}_{\infty }}={k}_{p}{t}^{n}$	Sy-St	0.947
		Sy-CS	0.824
		Sy-St-CS	0.955

While the Hixson–Crowell model was employed to demonstrate how the surface area of the carrier particles affected the releasing behavior^74^. Finally, the Korsmeyer–Peppas model is a very common model used for investigating the polymeric diffusion releasing mechanism^75^ where 60% of release data were fitted in Korsmeyer–Peppas model^76^. The representative equations of the Higuchi, Hixson–Crowell, and Korsmeyer–Peppas kinetic models could be represented, in Table 3^71,73,75.^

where W_t_ represents the released amount of active agent during time t; W_o_ is the initial concentration of released active agent (generally, W_o_ = 0); K_0_ is the zero-order constant; and K_1_ is the first-order constant; W_∞_ is the amount of fertilizer released at infinite time; K_h_ is the Higuchi model rate constant; K_HC_ is the Hixson–Crowell model rate constant; and K_p_ is the Korsmeyer–Peppas model rate constant.

**Figure 8:**
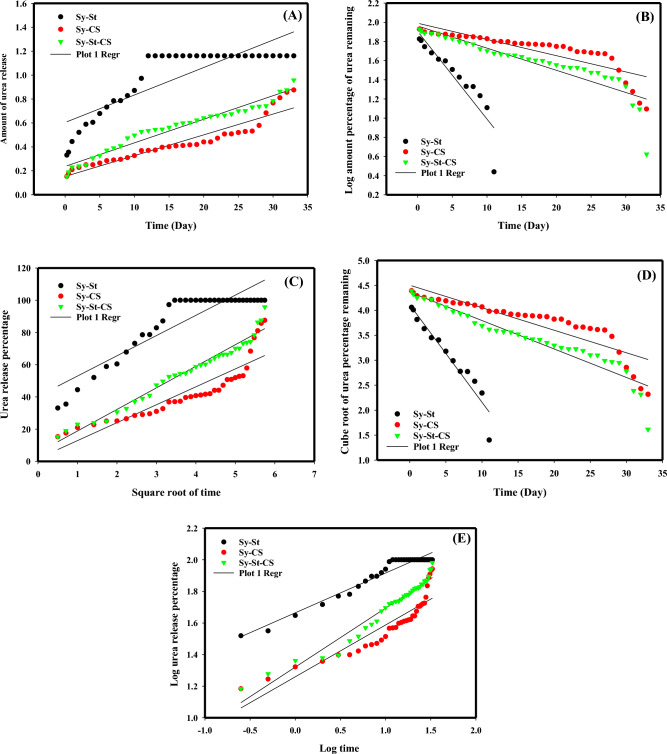
Kinetic models of urea release from Sy-St, Sy-CS, and Sy-St-CS, (**A**) zero-order, (**B**) first-order, (**C**) Higuchi, (**D**) Hixsonn˜ Crowell, and (**E**) Korsofmeyer–Peppas models. Suppl. Figure 2: ANOVA residual plots for release urea through Sy-St-CS hydrogel.

Using the example equations of the models, linear regression was used to fit the data to the examined models (Fig. 8A–E). Table 3 displays the computed mathematical theoretical parameters. The resulting coefficient (R^2^) values indicated that the zero-order model (0.893) better fit the release mechanism of urea from sample Sy-CS, indicating that urea concentration was not the controlling factor in the urea releasing rate during dissolution and diffusion from the matrix. The resulting coefficient (R^2^) values indicated that the Korsmeyer–Peppas model (0.947), which depicts the polymeric diffusion releasing mechanism, better fit the urea release mechanism from sample Sy-St. The sample Sy-St-CS hydrogel's coefficient (R^2^) values revealed values that were roughly in line with the Higuchi model (0.969), indicating that the releasing behavior of the urea from the matrix system occurred via dissolution and diffusion techniques.

### Biodegradation behavior of the hydrogels

Interestingly, the biodegradation of algae hydrogels has beneficial role for soil and agriculture. The algae matrix provides crops with protection from bacteria and insects^77–79^. Algae stimulate the plant for growth and development. Algae have biological properties that are antibacterial, anticarcinogenic, and antioxidant^80^. Algae are also known to be a naturally occurring source of bioactive compounds^81^. Among the physiologically active phytochemicals produced are carotenoids, terpenoids, xanthophylls, chlorophylls, phycobilins, polyunsaturated fatty acids, polysaccharides, vitamins, sterols, tocopherol, and phycocyanins. Algae are still viewed as undervalued resources on a global scale^82^.

**Figure 9:**
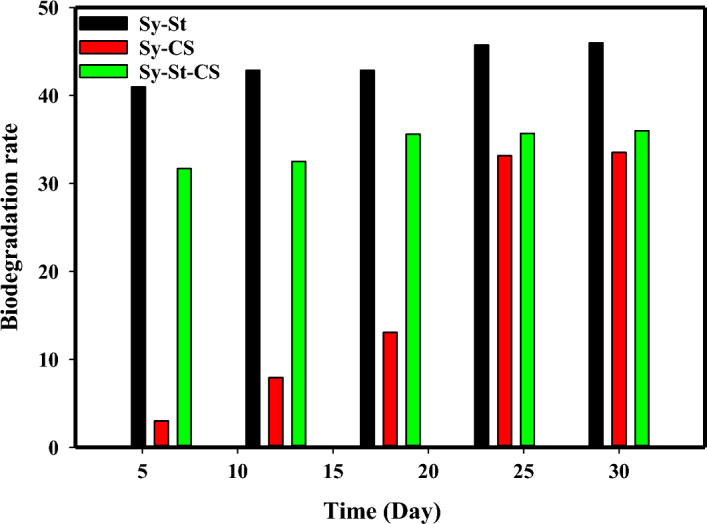
Biodegradation percentage from Sy-St, Sy-CS, and Sy-St-CS hydrogels.

The biodegradation of all studied hydrogels in soil are vital issue for their stability^83,84^. The biodegradation of hydrogels (Sy-CS, Sy-St, and Sy-St-CS) was assessed at RT for 30 days. Firstly, all hydrogels were degraded in soil with different ratios due to the active species of various microorganisms present in the compost and soil, which speed up the degradative process. The rate of degradation gradually elevated over time, as shown in Fig. 9. The degradation rates for the samples Sy-CS, Sy-St, and Sy-St-CS after 30 days were 33%, 45%, and 36%, respectively. The data depicted the rapid rate biodegradation of Sy-St Hydrogel from first 5 to 30 days. The rate was jumped from 40.5 to 45%, respectively. This behavior was due to presence of many hydroxyl groups water molecules impregnated to the matrix which weaken the hydrogel. Besides, The porous and layered nature of hydrogel, water and bacteria can seep out of them^85^. The hydrophilicity of hydrogel allows moisture from the soil to penetrate to the polymer network, weakening the polymer chains in the process^86,87^. However, the rate of Sy-CS and Sy-St-CS hydrogels were slower than starch hydrogel only. The primary cause of long term of hydrogel retention in soil is its encapsulation through chitosan. It also is the most significant for boosting plant defense and phage inactivation^88^. Evidently, chitosan may be used for induction of phago resistance in industrial microorganism cultures to prevent undesirable phagolysis caused by inoculum contamination by virulent bacteriophages or by spontaneous prophage induction in lysogenic cultures^89^.

### Water-holding capacity (WHC) in soil

The retention of large amount of water in soil is the vital target for agriculture. It facilitates diffusing the algae encapsulated in hydrogel and can improve the water holding of soil through binding it with various water-soluble compounds in their structures. In the bare soil, the water-holding capacity was (33.62%), but after blending small amount of hydrogels nearly 0.25%, the capacity increased to (99.96%), (99.44%), and (101.5%) by using Sy-CS, Sy-St, and Sy-St-CS, respectively.

### Water retention in soil

**Figure 10:**
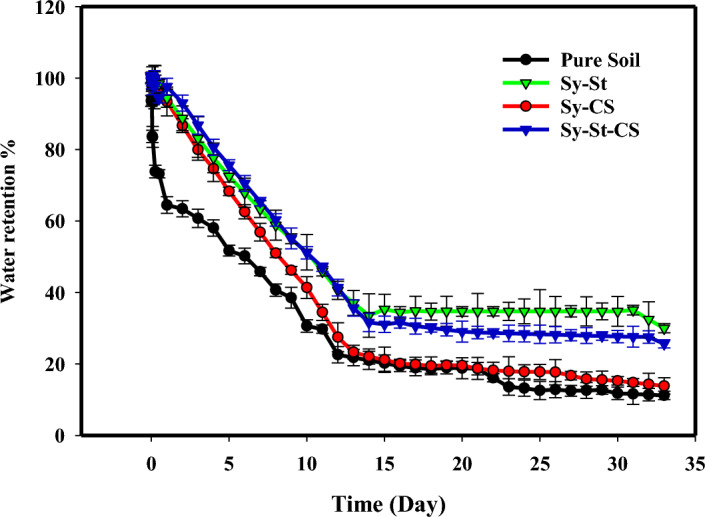
Water retention in soil percentage from Sy-St, Sy-CS, and Sy-St-CS hydrogels.

Evaluating the capacity for water retention in soil is a crucial issue for explaining the potential of the hydrogel in soil. Figure 10 demonstrates water retention for different soils. In bare soil without hydrogels, a fast decline in its capacity after the first 3 days was observed during the first irrigation. While the presence of hydrogels in soil was positively affected, the soil samples containing hydrogel became moist, which was evident for water uptake through soil. In this case, Sy-St-CS hydrogel exhibited a superior capacity for water retention than bare soil; it retained 40% of its capacity. This may be explained through a diffusion mechanism. The humidity in the soil decreased, and the water absorbed by the hydrogel was slowly released^90^. Consequently, soil containing the hydrogel could possess more moisture during irrigation periods than others. Following this, water would be gradually released from the hydrogel at any time of subsequent dryness^1,90^.

### Reswelling capacity (RSC) in soil and water

**Figure 11:**
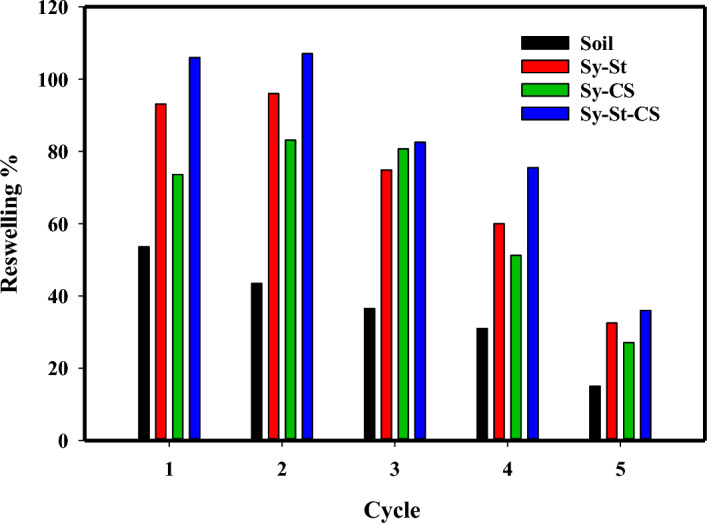
Reswelling capacity in soil percentage from Sy-St, Sy-CS, and Sy-St-CS hydrogels during five cycles.

The capacity of hydrogels for reswelling water from soil through the number of swelling-drying cycles was investigated at Fig. 11. Four measurements were applied for assessment the soil ability to retain water. For each measurement, moist soil dried to a constant weight. Noticeably, after each drying cycle, the modified soil with the hydrogel has significant ability for water absorption than bare soil. There were differences in water retention for each swelling-drying cycle. Initially, the first and second cycles achieved superior water retention. However, there was a decline in water retention at progressive cycles. This decrease was due to the partially deterioration of hydrogels. In addition to change in the ionic osmotic pressure, may have contributed to the decrease in water absorption capacity^10^. At first cycle, the hydrogels exhibit high percentage of retention of 105.6%, 309% and 794% for Sy-CS, Sy-St, and Sy-St-CS, respectively. However, the ability of the hydrogel for holding water gradually diminished and the equilibrium water absorbency is decreased consecutively. After five cycles, the ability was further decreased. For Sy-CS, Sy-St, and Sy-St-CS, the value were found to be (55%), (194%), and (390%), respectively.

Finally, Table 4 illustrates the comparison between different hydrogels based on chitosan and starch for representing the release efficiency of hydrogels compared to studied hydrogels.

**Table 4:** The comparison between hydrogels based biopolymer for release capacity.

Based materials	Release %	Time	References
Macrospheres prepared with chitosan and chitosan-starch blends by an easy dripping technique, using a sodium tripolyphosphate aqueous solution as the crosslinking agent	release medium reached between 70 and 93%	After 14 days	^22^
Composite of chitosan-modified smectite clays consist of montmorillonite (MMt) and saponite (SAP) clay minerals and their urea adsorption–desorption study	50–60%	5 days	^91^
Fertilizer formulation, based on urea incorporating chitosan (CS) and poly (vinyl alcohol) (PVA) blend	22%	10 days	^92^
Synthesis of chitosan—starch coated fertilizers	30%:70%	168 h	^93^

## Conclusions

This present study was conducted to fabricate a hydrogel based on microalgae with some biopolymers, such as chitosan and starch, together. Their functional, morphological, and textural properties were investigated. These biohydrogels can be used as an effective substrate for urea fertilizer. All optimisation processes were studied for effective urea substrates, including hydrogel swelling rate, loading, urea release, water-holding capacity, water retention, and re-swelling capacity in soil and water properties. This study introduces a suitable method for designing Sy-CS-St with efficient slow urea release, water retention, and water-holding capacity properties. Urea loading on Sy-CS-St hydrogels depended on the swelling degree of hydrogels, so the maximum loading percentages of samples Sy-St-CS hydrogel (465%), Sy-St hydrogel (440%), and Sy-Cs hydrogel (99%) at 9 M urea concentration The urea release behaviour reached equilibrium after 30 days, and the kinetic data showed the fitting of Sy-St-CS hydrogel with the Higuchi model, Sy-St fitting with the korsmeyer–Peppas model, and Sy-CS fitting with the Zero-order model. Incorporating a small amount of low-cost Sy-St-CS hydrogel in the soil improved the water-holding capacity significantly. Impregnation of the hydrogel enhances the porosity of the soil, which facilitates the growth of the plant through seed germination. Therefore, the introduction of Sy-St-CS-Urea hydrogels in soil becomes a good candidate fertilizer to improve water retention, reduce urea loss, inhibit the growth of harmful microbes, and improve crop yield. This study is recommended to be applied in soil in the further study. Future investigations may focus on enhancing formulations tailored to particular crops, investigating potential collaborations with other agricultural inputs, and evaluating the prolonged impacts in various soil conditions.

### Supplementary Information

Supplementary Information.

## Data Availability

All data generated or analysed during this study are included in this published article.

## Abbreviations

3D: Three-dimensional matrices
W_∞_: The amount of fertilizer released at infinite time
b: The Langmuir constant
_Ce_: The equilibrium concentration of urea
CRFs: Controlled-release fertilizers
CS: Chitosan
FTIR: Fourier transform infrared spectroscopy
K_f_& n: Freundlich isotherm constants
Q_0_: The highest monolayer adsorption rate
q_e_: The amount of urea adsorbed per mg of the adsorbent
R^2^: The coefficient
RSC: Reswelling capacity
RT: Room temperature
SRF: Slow release fertilizer
SRFH: Slow release fertilizer hydrogel
St: Starch
Sy: *Synechocystis* sp.
WHC: Water-holding capacity
WM: Wuxal media
WR: Water retention
XRD: X-ray diffraction
K_0_: Zero-order constant
K_1_: First-order constant
K_h_: Higuchi model rate constant
K_HC_: Hixson–Crowell model rate constant
K_p_: Korsmeyer–Peppas model rate constant
