# Author Correction: Urea intercalated encapsulated microalgae composite hydrogels for slow-release fertilizers

**DOI:** 10.1038/s41598-024-68544-y

**Published:** 2024-07-31

**Authors:** Nada Sarhan, Esraa G. Arafa, Nada Elgiddawy, Khaled N. M. Elsayed, Fatma Mohamed

**Affiliations:** 1 Department of Biotechnology and Life Sciences, Faculty of Postgraduate Studies for Advanced Sciences (PSAS), Beni-Suef University, Beni-Suef, 62 511 Egypt; 2 Chemistry Department, Faculty of Science, Beni-Suef University, Salah Salim St, Beni-Suef, 62514 Egypt; 3 Botany and Microbiology Department, Faculty of Science, Beni-Suef University, Beni-Suef, 62511 Egypt; 4 Materials Science Research Laboratory, Chemistry Department, Faculty of Science, Beni-Suef University, Beni-Suef, 62514 Egypt; 5 Nanophotonics and Applications Lab, Faculty of Science, Beni-Suef University, Beni-Suef, 62514 Egypt

Correction to: *Scientific Reports* 10.1038/s41598-024-58875-1 published online 01 July 2024.

The original version of this Article contained an error in the spelling of the author Nada Elgiddawy, which was incorrectly given as Nada Elgedawy.

The original Article has been corrected.

