# Supplementary material for: Urea intercalated encapsulated microalgae composite hydrogels for slow-release fertilizers
Source: Sci Rep. 2024 Jul 1;14:15032. doi: 10.1038/s41598-024-58875-1 (PMC11217492; doi:10.1038/s41598-024-58875-1)
Supplement: Supplementary file 1 — Supplementary Information. [file 41598_2024_58875_MOESM1_ESM.docx]

**Supplementary Material**

**Suppl.** **Table 1**: Residuals Statistics of Freundlich model for urea loading through Sy-St- CS hydrogel

|  | Minimum | Maximum | Mean | Std. Deviation | N |
| --- | --- | --- | --- | --- | --- |
| Predicted Value | -.03854992 | .51194733 | .26557616 | .224766260 | 5 |
| Std. Predicted Value | -1.353 | 1.096 | .000 | 1.000 | 5 |
| Standard Error of Predicted  Value | .037 | .064 | .049 | .011 | 5 |
| Adjusted Predicted Value | -.09492517 | .47573721 | .24868156 | .234882271 | 5 |
| Residual | -.118841983 | .048353098 | .000000000 | .068290710 | 5 |
| Std. Residual | -1.507 | .613 | .000 | .866 | 5 |
| Stud. Residual | -1.701 | .741 | .080 | 1.030 | 5 |
| Deleted Residual | -.151445717 | .085715167 | .016894599 | .098903176 | 5 |
| Stud. Deleted Residual | -7.405 | .669 | -1.109 | 3.528 | 5 |
| Mahal. Distance | .061 | 1.831 | .800 | .709 | 5 |
| Cook's Distance | .001 | .397 | .225 | .171 | 5 |
| Centered Leverage Value | .015 | .458 | .200 | .177 | 5 |

Histogram

Dependent Variable: logqe

1

0

-1

-2

Frequency

3

2

1

0

Mean =

7.08E-16

Std. Dev. =

0.866

N =

5

Regression Standardized Residual

Normal P-P Plot of Regression Standardized Residual

Dependent Variable: logqe

1.0

0.8

0.6

0.4

0.2

0.0

Expected Cum Prob

1.0

0.8

0.6

0.4

0.2

0.0

Observed Cum Prob

Scatterplot

Dependent Variable: logqe

1.5

1.0

0.5

0.0

-0.5

-1.0

-1.5

Regression Standardized Residual

1.0

0.5

0.0

-0.5

-1.0

-1.5

-2.0

y=7.08E-16+9.71E-16*x

R

2

Linear =

0

Regression Standardized Predicted Value

**Suppl.** **Figure 1:** ANOVA residual plots for urea loading through Sy-St-CS hydrogel.

**Suppl.** **Table 2:** Residuals Statistics of Higuchi model for urea release through Sy-St- CS hydrogel.

|  | Minimum | Maximum | Mean | Std. Deviation |
| --- | --- | --- | --- | --- |
| Predicted Value | .241823182 | .887098372 | .553131501 | .2002844220 |
| Std. Predicted Value | -1.554 | 1.667 | .000 | 1.000 |
| Standard Error of Predicted  Value | .007 | .014 | .010 | .002 |
| Adjusted Predicted Value | .251691997 | .878308892 | .553699766 | .1995824181 |
| Residual | -.0891873538 | .0725718811 | .0000000000 | .0421437316 |
| Std. Residual | -2.085 | 1.696 | .000 | .985 |
| Stud. Residual | -2.197 | 1.756 | -.006 | 1.019 |
| Deleted Residual | -.0990561917 | .0796515644 | -.0005682645 | .0451353214 |
| Stud. Deleted Residual | -2.342 | 1.816 | -.008 | 1.040 |
| Mahal. Distance | .000 | 2.780 | .971 | .866 |
| Cook's Distance | .000 | .267 | .036 | .056 |
| Centered Leverage Value | .000 | .082 | .029 | .025 |

Histogram

Dependent Variable: Urea

2

1

0

-1

-2

-3

Frequency

10

8

6

4

2

0

Mean = -2.28E-15

Std. Dev. = 0.985

N = 35

Regression Standardized Residual

Normal P-P Plot of Regression Standardized Residual

Dependent Variable: Urea

1.0

0.8

0.6

0.4

0.2

0.0

Expected Cum Prob

1.0

0.8

0.6

0.4

0.2

0.0

Observed Cum Prob

Scatterplot

Dependent Variable: Urea

2

1

0

-1

-2

Regression Standardized Residual

2

1

0

-1

-2

-3

y=-2.28E-15-1.31E-15*x

R

2

Linear =

0

Regression Standardized Predicted Value

**Suppl.** **Figure 2:** ANOVA residual plots for urea release through Sy-St -CS hydrogel.
